# Biological effects of Thymol loaded chitosan nanoparticles (TCNPs) on bacterial plant pathogen *Xanthomonas campestris pv. campestris*

**DOI:** 10.3389/fmicb.2022.1085113

**Published:** 2022-12-22

**Authors:** Sarangapani Sreelatha, Nadimuthu Kumar, Sarojam Rajani

**Affiliations:** Temasek Life Sciences Laboratory, National University of Singapore, Singapore, Singapore

**Keywords:** metabolomics, nanoparticles, oxidative stress, spectroscopy, chitosan

## Abstract

Engineered nanomaterials can provide eco-friendly alternatives for crop disease management. Chitosan based nanoparticles has shown beneficial applications in sustainable agricultural practices and effective healthcare. Previously we demonstrated that Thymol loaded chitosan nanoparticles (TCNPs) showed bactericidal activity against *Xanthomonas campestris* pv *campestris (Xcc),* a bacterium that causes black rot disease in brassica crops. Despite the progress in assessing the antibacterial action of TCNPs, the knowledge about the molecular response of *Xcc* when exposed to TCNPs is yet to be explored. In the present study, we combined physiological, spectroscopic and untargeted metabolomics studies to investigate the response mechanisms in *Xcc* induced by TCNPs. Cell proliferation and membrane potential assays of *Xcc* cells exposed to sub-lethal concentration of TCNPs showed that TCNPs affects the cell proliferation rate and damages the cell membrane altering the membrane potential. FTIR spectroscopy in conjunction with untargeted metabolite profiling using mass spectrometry of TCNPs treated *Xcc* cells revealed alterations in amino acids, lipids, nucleotides, fatty acids and antioxidant metabolites. Mass spectroscopy analysis revealed a 10–25% increase in nucleic acid, fatty acids and antioxidant metabolites and a 20% increase in lipid metabolites while a decrease of 10–20% in amino acids and carbohydrates was seen in in TCNP treated *Xcc* cells. Overall, our results demonstrate that the major metabolic perturbations induced by TCNPs in *Xcc* are associated with membrane damage and oxidative stress, thus providing information on the mechanism of TCNPs mediated cytotoxicity. This will aid towards the development of nano- based agrochemicals as an alternative to chemical pesticides in future.

## Introduction

Crop pathogens and pests significantly affect the yield and quality of agricultural production, causing substantial economic loss affecting food security (Ristaino et al., 2021). An estimated 2 million tons of pesticides are used annually worldwide towards effective crop protection (Sharma et al., 2019). Even though such agrochemicals have helped to increase crop production, its extensive use damages the environment and leads to bio magnification in the ecosystem (Meena et al., 2020). To combat the risk posed by chemical pesticides, nanotechnology can offer suitable solutions. Use of nanomaterials for controlled release of pesticides, enhances its efficiency and reduces the amount used in field applications. Current research stresses the utility of nanoparticles prepared from natural resources for ensuring biocompatibility, biodegradability, and ecological safety (Bandara et al., 2020). Hence, eco-friendly crop protection strategies based on naturally derived nanomaterials are being developed. Chitosan is a natural polysaccharide derived from Chitin and chitosan nanoparticles are being extensively studied for applications in the field of agriculture, medicine and food packaging (Yanat, and Schroën, 2021). Due to its antimicrobial properties, chitosan based nanoparticles are suitable to be used as pesticides and for food preservation. Chitosan biopolymer has also been utilized as an encapsulating agent for controlled release of fertilizers and as carriers for reducing the toxicity of herbicides (Rashidipour et al., 2019). Nanochitosan along with bio inoculants are also used in agriculture field to boost beneficial microbial population to improve crop yield in maize and fenugreek plant and soil fertility (Kumari et al., 2020; Agri et al., 2021, 2022). Thymol is a volatile monoterpenoid phenol, which is an active ingredient of essential oils found in medicinal herbs. It has antimicrobial and antioxidant properties and shown to prevent post-harvest loss in agriculture (Singh et al., 2017). Antimicrobial effect of thymol loaded chitosan nanoparticles has been investigated against several food borne bacterial pathogens (Pecarski et al., 2014).

*Xanthomonas campestris pv campestris (Xcc)* is an important bacterial pathogen worldwide that causes black rot disease of cruciferous crops (Nuñez et al., 2018). Recently, we studied the antibacterial effect of TCNPs on *Xcc* cells and found that it involved disruption of the membrane, generation of intracellular ROS, and cell death (Sreelatha et al., 2022). Studies regarding the mechanism of antimicrobial activity of nanoparticles against pathogens shows that it is complex and involves processes like oxidative stress induction, metal ion release, and non-oxidative mechanisms that results in cell death (Yu et al., 2020). It is known that microorganisms modulate their metabolic composition when exposed to environmental conditions and antibacterial agents (Chang et al., 2012). Metabolomics and Fourier transform infrared (FTIR) spectroscopy can reveal the metabolic perturbations occurring in microbial cells in response to antibacterial agents. Understanding the reprogramming of bacterial cellular biochemical network is important to elucidate the mode of action of antibacterial agent and the possible bacterial adaptation mechanisms (Goodacre et al., 2004; Pradier et al., 2005). Hence, to decode the mechanism of antimicrobial activity of TCNPs against *Xcc*, there is a need to understand how TCNPs affects the metabolic process of *Xcc* cells. This will help to understand and corroborate the observed physiological responses of *Xcc* cells upon TCNP exposure.

In this study, we first expanded on the toxicity studies of TCNPs at sub-lethal concentration. Previously we showed that TCNPs can generate ROS in *Xcc* cells (Sreelatha et al., 2022).Generally, when ROS production is observed, the mode of action of nanomaterial is commonly attributed to cell membrane damage and oxidative stress (Leung et al., 2016). Lipid peroxidation and membrane potential studies were done to see if ROS produced can indeed result in cell membrane damage. Further, the metabolic changes induced in *Xcc* cells when exposed to TCNPs was investigated. Towards this, an untargeted profiling of metabolites by using mass spectrometry was performed. Additionally, FTIR spectra was obtained to explore the spectral changes in *Xcc* cells when treated with TCNPs. This combined metabolomics approach revealed that exposure to TCNP leads to significant perturbations in amino acids, lipids, nucleotides and carbohydrate metabolic pathways. Overall, our results demonstrate that TCNPs induce toxicity in *Xcc* cells most likely *via* the disruption of cell membrane and induction of oxidative stress, and that metabolomics can provide detailed insights on the mechanism of nanoparticle mediated toxicity.

## Materials and methods

### Chemicals and bacterial strain

The *Xcc* used in the current study was obtained from American Type Culture Collection (ATCC 33913) and cultured as per the protocol prescribed by ATCC. Initially bacteria were streaked from −80°C glycerol stock on a nutrient agar plate and single colony was inoculated into YGC (Yeast extract, glucose, and calcium carbonate) media and incubated at 28°C for 24 h. From there, 10^8^ CFU/ml bacterial cell suspensions were taken for all subsequent experiments. Nutrient agar, broth media, chitosan low molecular weight (LMW CS) (85% degree of deacetylation), thymol and sodium tripolyphosphate (TPP) were purchased from Sigma–Aldrich Co (MO, USA).

### Tryphan blue Dye exclusion assay

Selective staining with fluorescent dyes is a valuable tool for quantification of cell size. The tryphan blue dye exclusion assay was performed to measure the toxicity and confirm the damage of bacterial cell wall by TCNPs against *Xcc* by the method described in Bayade et al., 2021. To assess the toxicity of TCNPs, viable cell counting was carried out both with *Xcc* alone and with TCNPs treated at the sub lethal dose (500 μg/ml) by tryphan blue staining. The biocidal effect of TCNPs was measured as cell mortality (M) and it was calculated as M = (1-Cv/Ct) x 100, where Cv is the number of viable cells in the treated sample and Ct the number of viable cells in the control.

### Rapid bactericidal activity

Rapid bactericidal activity of thymol-loaded chitosan nanoparticles was determined by Time dependent growth inhibition assay and Time- killing assay as described by Yoshimasu et al. (2018). Briefly, 200 μl of bacterial cells (approximately 10^8^CFU/ml) was inoculated in each well with TCNPs at concentration of 500 μg/ml and incubated at 28°C and the optical density recorded at 2 h interval for up to 24 h at a wavelength of 600 nm. The growth rate was obtained by plotting a graph for turbidity against time denoting the bactericidal effects of TCNPs. This growth inhibition concentration documents the changes to the rate of growth over time to observe changes in the log phase and final optical density. The time -kill assay was performed by using the aliquots from the same samples which were serially diluted and incubated at 28°C for 48 h followed by CFU counting.

### Membrane potential assay

Membrane potential was measured using the Fluorescent reporter probe (3,3′-Diethyloxacarbocyanine, iodide) DiOC_2_ and carbonyl cyanide m-chlorophenyl hydrazine (CCCP) as a positive control (Hudson et al., 2020). Briefly, bacterial culture was grown to the exponential log phase (10^8^ CFU/ml) and then incubated with and without TCNPs (500 μg/ml) for 24 h. After incubation, cells were diluted with PBS (to ~1 × 10^6^ cells/ml). DiOC_2_(3) working solution (10 μl) was added to each sample, and incubated for 5–30 min and the fluorescence was recorded in the Teccan Microplate reader using 450-nm excitation. Red fluorescence intensity was recorded at 670-nm emission.

### Lipid peroxidation assay

The Lipid peroxidation (LPO) potential of TCNPs was investigated according to the established protocol (Hossain et al., 2019). Briefly, 1 ml of each of the bacterial cultures were mixed with 200 μl (i.e., 500 μg/ml) of each of the TCNPs and incubated at room temperature. After centrifugation, 2 ml of 10% TCA was mixed with the TCNP-treated *Xcc* culture and centrifuged at 11,000 g for 35 min at room temperature to separate the insoluble cellular components. The supernatant was taken out and centrifuged again in the same relative centrifugal force (g) for 20 min to remove any protein precipitates as well as dead cells. Finally, the supernatant containing malondialdehyde was collected in a fresh tube, and mixed with freshly prepared thiobarbituric acid (TBA) solution and incubated in a hot water bath for 10 min to facilitate the formation of malondialdehyde-TBA adduct before cooling down to room temperature. The absorbance of the malondialdehyde-TBA adduct was measured by Teccan microplate reader at 532 nm.

### FTIR based bioassay

Briefly, bacterial culture (10^8^ CFU/ml) was incubated with 500 μg/ml of TCNPs for 6 h and then it was centrifuged at 10,000× g for 10 min at 4^o^ C and the supernatant was discarded. The cell pellet was harvested at the exponential phase (6 h) to assess the influence of TCNPs against *Xcc* cells. The pellet was washed three times with physiological saline and then dried in a desiccator at 45 ^o^ C at a constant dry weight. For FTIR analysis, the dried samples were powered in small agate mortar for 5 min and used for further measurements (Roscini et al., 2010). FTIR measurements were performed using a Nicolet 6,700 FTIR spectrometer (Thermoscientific Corporation, USA). Spectra were collected with 256 scans (resolution 4 cm^−1^) each and manipulated using OMNIC software (version-5). Baseline was corrected for each spectrum using the “automatic baseline correction” and then the spectra was smoothed. For each sample a total of 50 spectra was collected. All the FTIR measurements were repeated six times for each sample to be reproducible.

### Metabolomics analysis by LCMS

Metabolomics analysis was used to determine the differential metabolites of bacteria when exposed to TCNPs. Briefly, bacterial culture (10^8^ CFU/ml) was incubated with 500 μg/ml of TCNPs for 6 h to investigate the changes in the primary metabolism and then it was centrifuged at 10,000× g for 10 min and the supernatant was discarded. The pellet was rinsed twice with phosphate buffered saline to remove any residual media. The suspended cells were then centrifuged to form a pellet and the supernatant was discarded. To the cell pellets, 0.5 ml of methanol was added at −80°C followed by 1.5 ml of 50% methanol:H_2_0 at 4°C. The cells were lysed completely, and the crude extract was centrifuged at 48,000 g for 1 h, then the supernatant was collected. An aliquot of 500 μl of supernatant was filtered with 3 kDa Nanosep filter and then centrifuged at 14,000 g, The filtrate was collected, vacuum dried, and held at ≤ −80°C until analysis. The dried sample was resuspended in 1 ml methanol and used for further metabolites analysis as described in Huang et al. (2019).

Two microliter of the concentrated extracts were injected into Accucore RP-MS C18 column (2.1 × 100 mm, 2.6 μm particle size; Thermo scientific, Breda, Netherlands) with 0.3 ml/min flow rate for LC-ESI-MS/MS analysis of metabolites independently. The mobile phase (A) composed of 0.1% formic acid in water and 100% methanol (B). The elution gradient started with 5% methanol for 1–5 min, then increased linearly to 100% at 10–18 min, and then decreased to 5% at 20.0 min. The oven temperature was maintained at 40°C. Detection was performed in both positive and negative mode with full MS scan in the mass range 70–950 *m/z* at a resolution of 70,000 and data dependent MS/MS (ddMS^2^)using a Thermo Orbitrap Q –Exactive quadrupole mass spectrometer. The MS parameters included drying gas temperature, 350°C; gas flow rate, 12 l/min; nebulizer pressure, 35 psi; sheath gas temperature, 400°C; sheath gas flow rate, 12 l/min; delta electron multiplier volt, 500 V; and spray voltage 3.8 kV. Xcalibur software (version 5.0, Thermo) was used to control the instrument and to acquire the MS data. The raw data acquired by Xcalibur software was then processed by compound discoverer software (version 5.0, Thermo) for peak alignment,peak detection and peak integration.All data were normalized to sum of metabolites for each sample. Compound discoverer software was used for peak annotation after data processing with public databases like chem spider, m/z cloud and METLIN. Confirmation of these putative hits was done by MS2 fragment spectra comparison with the public databases. Principal component analysis (Supplementary Figure S1) was used for the classification of the samples. Student *t*-tests and fold changes were used to compare the concentration of metabolites between the samples.

### Data analysis

All the parameters studied were subjected to statistical treatment using SPSS statistical package (Version 23.0). Data are represented as mean ± S.D. of three independent experiments, each performed in triplicates. One-way ANOVA was adopted to all the parameters under study to test the level of statistical significance. The difference was considered significant if *p < 0.05.

## Results and discussion

### Antimicrobial activity and time kill kinetics of TCNPs

**Figure. 1:**
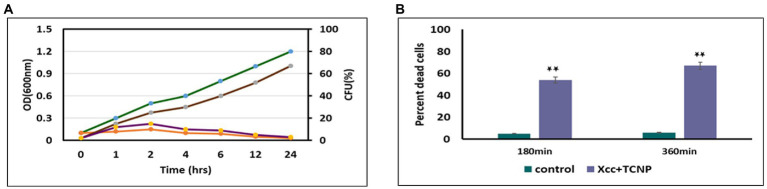
**(A)** Effect of TCNPs (500 ug/ml) at sublethal dosage for 24 h on *Xcc* cell growth measured through OD (green and orange curves for control and TCNP treated *Xcc* cells) and CFU (brown and violet for control and TCNP treated *Xcc* cells); **(B)** percent dead cells when exposted to TCNP nanoparticles by tryphan blue staining. Data are represented as mean ±. S.D. of three independent experiments, each performed in triplicates and considered statistically significant when *p < 0.05.

In our previous study, we observed that TCNPs exhibited strong bactericidal activity against *Xcc.* This was demonstrated in terms of decrease in *Xcc* growth and viability with different concentrations of TCNPs (Sreelatha et al., 2022). The time kill kinetic and tryphan blue dye exclusion assays were used to investigate the cytotoxicity and damage caused by TCNPs at a sub-lethal concentration of 500 μg/ml to *Xcc* cells. TCNPs were found to cause cell mortality in a time dependent manner. When compared to control, the TNCP treated *Xcc* cells, displayed reduced number of bacterial colonies within an hour and by 24 h showed complete absence of growth (Figure 1A). The tryphan blue dye can penetrate cells with disrupted cytoplasmic membrane and used to quantify the cell death. TCNPs treated *Xcc* cells at 3 h and 6 h showed a significantly larger number of stained dead cells when compared to control cells (Figure 1B). The results demonstrate that TCNPs treatment causes cell membrane damage on *Xcc* cells leading to cell death. It is suggested that the lipophilic moieties in chitosan and thymol interact and damages the lipid bilayer of the bacterial cell membrane, increasing the membrane permeability (Liolios et al., 2009). Thus, TCNPs disrupts the cell membrane and provides a synergy of action leading to cytotoxicity.

### TCNPs reduces membrane potential and induce lipid peroxidation

**Figure 2:**
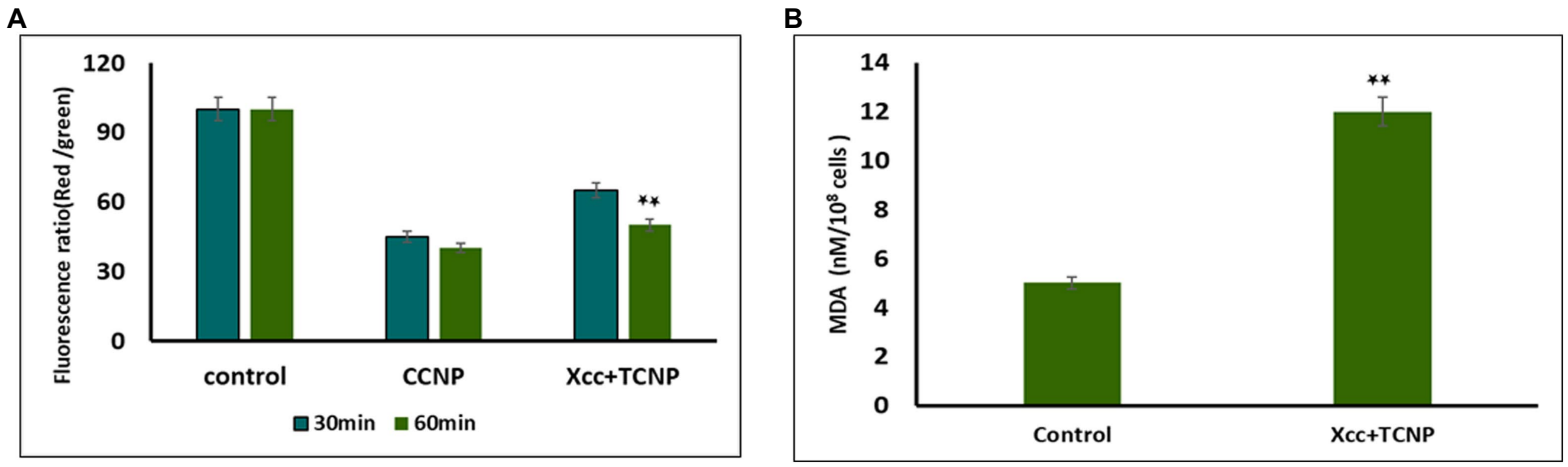
**(A)** Membrane potential of *Xcc* when exposted to TCNP nanoparticles at the sub-lethal dose. **(B)** Lipid peroxidation for *Xcc* cells when exposted to TCNP nanoparticles at the sub-lethal dose. Data are represented as mean ±. S.D. of three independent experiments, each performed in triplicates and considered statistically significant when *p < 0.05.

Studies have shown that membrane permeability in cells occurs mainly due to depolarization of active membrane potential (Chang et al., 2012; Khan et al., 2020). We used the fluorescent dye DiOC_2_(3) to examine the membrane potential changes in TNCP treated *Xcc* cells. DiOC_2_(3) is a positively charged membrane potential-sensitive dye that is used for ratiometric measurements of bacterial membrane potential. An increasing membrane potential leads to shift in fluorescence of DiOC _2_ (3) from green to red (Warren and Payne, 2015). Membrane potential was also measured for proton ionophore m-chlorophenylhydrazone (CCCP) following exposure to the cells, which is known to eliminate the proton gradient across the membrane. A lower ratio of red/green fluorescence was found in cells treated with CCCP. Upon addition of TCNPs, the *Xcc* cells also exhibited (Figure 2A) a decrease in red fluorescence with increased green fluorescence, resulting in a lower ratio of red/green fluorescence when compared to control cells.

This result indicates that at sub lethal concentrations, the TCNPs exposure leads to membrane depolarization. The hydroxyl group present on thymol is known to play an active role in depolarizing membrane potential (Xu et al., 2008). Membrane potential is a key to the survival of bacteria as it plays an essential role in regulating membrane permeability, ion homeostasis, organelle function, ATP synthesis and motility (Warren and Payne, 2015). Many studies have documented that bactericidal agents like antibiotics work by altering the membrane potential of bacterial cells (Hancock and Rozek, 2002; Chang et al., 2012). Therefore, the results highlighted the fact that the charged groups in chitosan and thymol binds to the negatively charged bacterial cell membrane causing disruption of the cell and thus increasing the membrane permeability (Liolios et al., 2009).

Our previous study showed that TCNPs results in intracellular ROS in *Xcc* cells, damaging the cellular organelles (Sreelatha et al., 2022). Cellular accumulation of ROS leads to lipid peroxidation (LPO), a key mechanism responsible for the increase in cell membrane permeability. The production of malondialdehyde (MDA) is a useful marker to monitor LPO. To determine if the increase in ROS generation in the treated *Xcc* cells leads to the increase of LPO, we measured the cellular production of MDA. MDA level was significantly increased in the *Xcc* cells after the exposure to the TCNPs showing that ROS mediated membrane lipid oxidation contributes to the antibacterial effect of TCNPs (Figure 2B). ROS radicals like OH and _1_O^2^ can initiate lipid peroxidation, resulting in severe structural changes in bacterial outer layer, including changes in its permeability, fluidity, and damage to the building components (Chang et al., 2012; Khan et al., 2020). Studies have shown that during lipid peroxidation, expression of apoptotic genes are stimulated leading to cell death (Wang et al., 2016). The above results indicate that TCNPs generated ROS leads to lipid peroxidation leading to increase permeability and facilitating the entry of TCNPs into the cells.

### FTIR analysis

**Figure 3:**
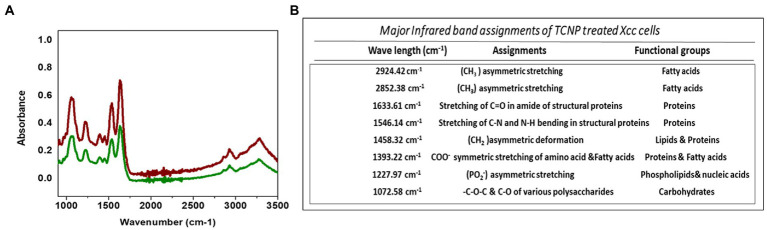
**(A)** ATR-FTIR spectra of control *Xcc* cells(Red line) and TCNP treated *Xcc* cells (green line). **(B)** Major assignment of FTIR peaks fot TCNP treated *Xcc* cells.

FTIR spectroscopy was performed to elucidate the structural changes in cellular composition of *Xcc* cells after exposure to TCNPs. Figures 3A,B shows the FTIR spectra collected between the region 1000–3500 cm^−1^ from control and treated *Xcc* cells with the IR signals derived from lipids, proteins, carbohydrates and nucleic acids. The spectra obtained from the TCNP treated *Xcc* cells showed a similar profile when compared to control cell but exhibited an overall decrease in intensity with few vibrational shifts to lower wavelengths. Similar overall decrease in the intensity of IR absorption signals were also observed in bacteria exposed to AgI/TiO2 for 6 h and in bacteria exposed with CDTe quantum dot nanoparticles (Hu et al., 2007; Fang et al., 2012).

**Figure 4:**
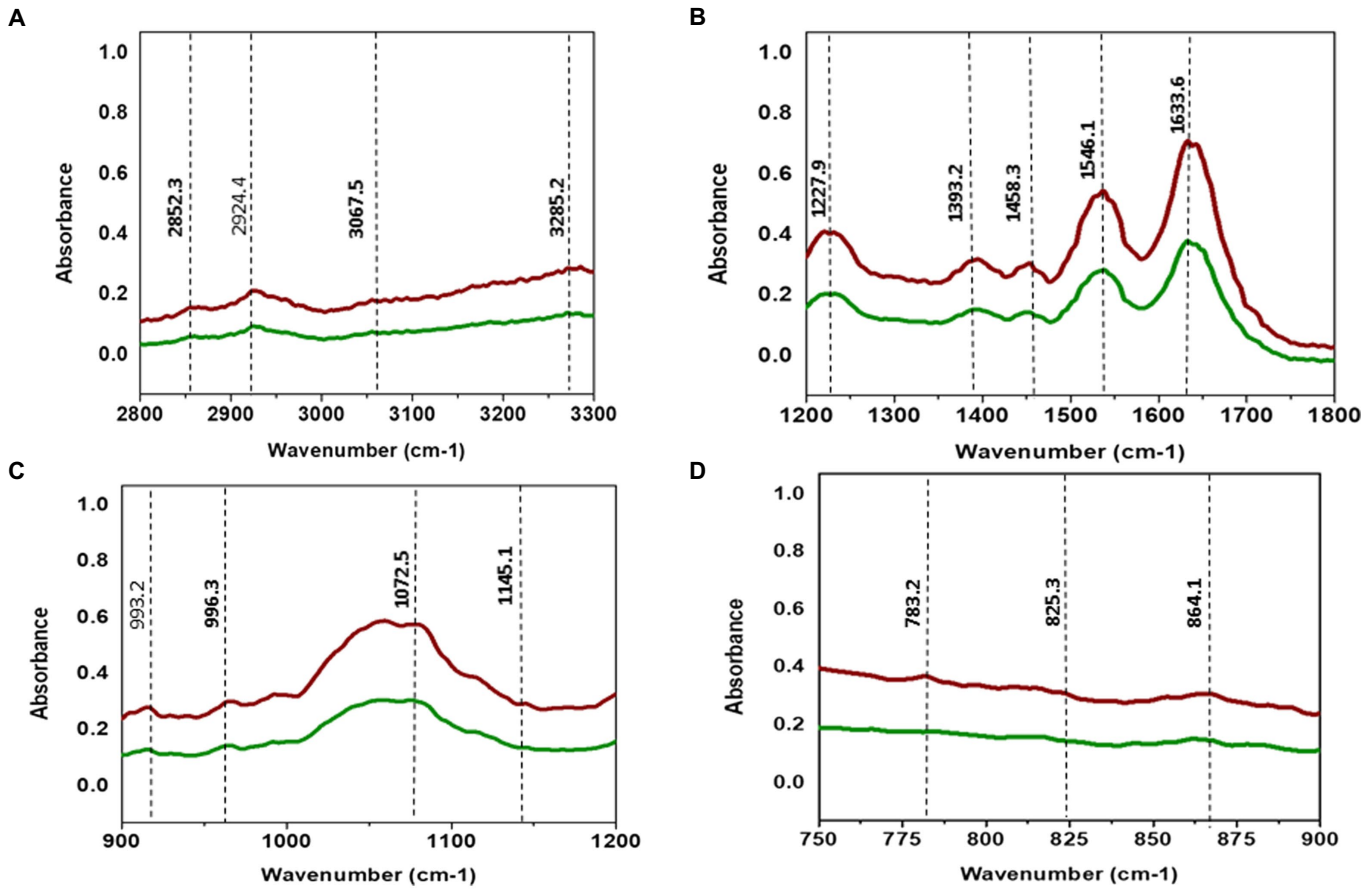
**(A)** ATR-FTIR spectra of control *Xcc* cells (Red line) and TCNP treated *Xcc* cells (green line). **(A)** Fatty acid region 2800–3300 cm^-1^. **(B)** 1200–1800 cm^-1^. **(C)** 900–1200 cm^-1^. **(D)** 700–900 cm^-1^.

TCNP treated *Xcc* cells showed a decrease in the intensity of peaks at 2924, and 2852 cm^−1^. The spectral bands between 3000–2800 cm^−1^ (Figure 4A) are associated with stretching vibrations of CH_2_ and CH_3_ functional groups in fatty acids and to a lesser extent, to the N-H amide group in proteins (Mariey et al., 2001). *E. coli* treated with CdTe quantum dot nanoparticles and ZnO (Zinc oxide) nanowire showed similar changes with decreased band intensity in the region between 2852–2959 cm^−1^ indicating changes in -CH vibrations of the fatty – tail structure. Modifications of -CH vibrations has been shown to represent changes in membrane fluidity of bacterial cell wall and cell lysis (Wang et al., 2011; Fang et al., 2012). The reduced peak intensity observed at 2924, 2852 cm^−1^ in TCNP treated *Xcc* cells indicates that the nanoparticle exposure alters the fatty acids in *Xcc* cell membrane making the membrane permeable and facilitating the entry of TCNPs into the cells.

The absorption bands obtained between 1800 and 1200 cm^−1^ (Figure 4B) are associated with amide I and amide II bands of proteins and peptides (Kiwi and Nadtochenko, 2005). TCNP treated *Xcc* cells showed reduced peak intensity at 1633 and 1546 cm^−1^. Peaks at these regions arise due to the stretching of C=O groups at α-helical and β-pleated sheet of proteins and stretching of N-H bending and C-N stretching vibrations of the peptide groups, respectively, (Kiwi and Nadtochenko, 2005). Hu et al. (2007) reported a decrease in peak intensity at 1653 and 1545 cm^−1^ when pathogenic bacteria was exposed to AgI/TiO2 nanoparticles and associated this with a reduction in concentrations of amide I and amide II groups.

The absorption peak at 1458 cm^−1^ in the TCNP treated *Xcc* cells showed a reduced peak intensity with a vibrational shift to a lower wavelength compared to the control cells at 1457 cm^−1^. Similar changes were observed in sublethally stressed *Listeria innocuo* with acetic acid at the peak around 1485 cm^−1^ due to the CH_2_ deformation of proteins and lipids (Wu et al., 2013). The intensity decrease at 1393 cm^−1^ and 1227 cm^−1^ peak in TCNP treated *Xcc* cells denotes changes in C=O symmetric stretching of COO- group in amino acids and fatty acids and in asymmetric stretching of (PO_2_^_^) phosphodiester bond in phospholipids, respectively. Similar spectral changes at 1386 cm^−1^ and 1226 cm^−1^ peaks were also observed in *E. coli* treated with CdTe quantum dot nanoparticles (Fang et al., 2012). Overall, the changes in the peak intensity between 1800- 1200 cm^−1^ region denotes that exposure to TCNPs results in damage or conformational/compositional alteration in proteins and lipids which can be components of cell wall peptides or intracellular proteins.

Spectral changes were also observed in the region 1200-900 cm^−1^ which indicates C-O-C and C-O ring vibrations of carbohydrates (Kiwi and Nadtochenko, 2005).

Decreased peak intensity with vibrational shifts were observed at 1145, 1072,993 and 966 cm-^1^ as shown in the Figure 4C in the treated *Xcc* cells. The changes observed at these peaks possibly reflect weakening of the C-O band due to peroxidation of lipopolysaccharides. Bacteria when exposed to ZnO nanoparticles reported similar changes in the spectral bands around 1200-900 cm^−1^ suggesting changes in carbohydrates due to ROS induced damage (Jiang et al., 2010).

The IR region between 900 and 600 cm^−1^ (Figure 4D) is referred to as the fingerprint region and contains weak bands corresponding to aromatic ring vibrations. Both control and TCNP treated *Xcc* cells displayed weak spectral features in this region. Similar weak spectral features in the 900-600 cm^−1^ region were also observed in the gram-negative bacteria both in the control and treated when exposed to carbon nanoparticles (Riding et al., 2012). Overall, the FTIR spectra revealed biochemical alteration to lipids, structural proteins, carbohydrates and nucleic acids in TCNP treated *Xcc* cells. These alterations can be attributed to damaged cell membrane and ROS generated oxidative stress upon TCNP exposure. The results from FTIR are consistent with the findings of (Sreelatha et al., 2022) which showed increased membrane permeability and ROS generation in TCNP treated *Xcc* cells.

**Figure 5:**
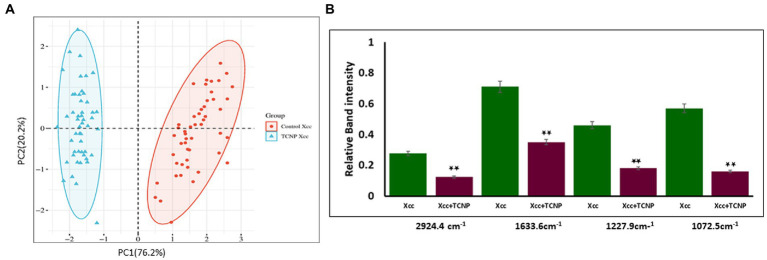
**(A)** PCA analysis of FTIR spectra. **(A)** PCS score plots showing distinct clustering between the control cells (Red dot) and treated *Xcc* cells (Blue dot). **(B)** Relative band intensities at the peak 2924 cm^-1^, 1633 cm^-1^, 1227 cm^-1^, and 1072 cm^-1^ are represented as mean ± S.D. of three independent experiments, each performed in triplicates and considered statistically significant when *p < 0.05.

Principal component analysis is an unsupervised method primarliy used to reduce the dimension of the data variables and to gain information of the spectra variation among the samples (Yang et al., 2021). PCA was applied to the spectral data set to identify whether any clustering and discrimination could be observed between the group. The spectra showed a possible separation between the control cells and TCNP treated *Xcc* cells. The score plot obtained as shown in Figure 5A denotes that the groups were separated along PC1 (76.2%) and PC2 (20.2%) which explains the spectral differences between the groups. To further confirm the spectral data, relative band intensity for lipids, nucleic acids,proteins and polysaccharides were calculated between the region 3000-1000 cm^−1^. TCNP treated *Xcc* cells showed lower absorption signals in the region 3000-1000 cm^−1^. The relative intensity at 2924 cm^−1^, 1633 cm^−1^,1227 cm^−1^, and 1072 cm^−1^ peaks were the most significantly reduced band signals in the treated *Xcc* cells than in the control cells as shown in Figure 5B.

### Significant changes in metabolites are observed after TCNP treatment.

**Figure 6:**
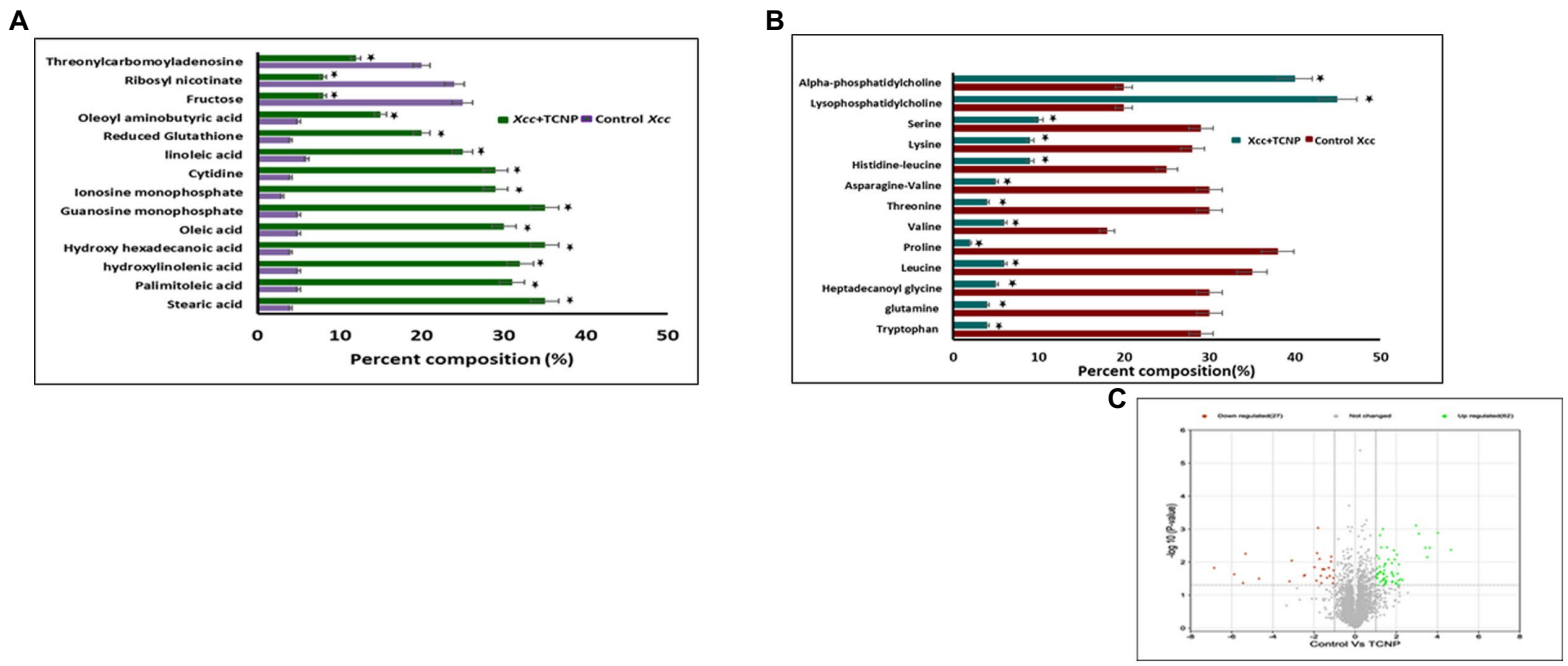
**(A)** Bar graph of top 27 differential metaboloites in the control and TCNP treated *Xcc* cells for fatty acids, nucleic acids, sugars etc. **(B)** Bar graph of metabolic response the control and TCNP treated *Xcc* cells for amino acids and lipids etc. Data bars indicate increased/decreased percentage of metabolite levels in treated *Xcc* cells. **(C)** Volcano plot showing upregulation and down regulation of metabolites. Values are means of 5 biological replicates. Data bars represent increased/decreased percentage percentage of metabolite levels in treated *Xcc* cells and considered statistically significant when *p < 0.05.

To investigate the changes in the metabolite composition of *Xcc* cells, when treated with TCNPs, we performed untargeted metabolomics analysis by mass spectrometry. Monitoring the metabolic variations in microorganisms shows the physicochemical state of the cells in response to stress (Liu et al., 2017). As shown in Figures 6A–C the metabolome of treated *Xcc* cells was significantly altered when compared to control cells. The most predominant metabolites that changed were amino acids, fatty acids and nucleotides as shown in the Supplementary Table S1. A significant decrease in abundance of amino acids like tryptophan, glutamine, leucine, proline, valine, threonine, asparagine, histidine lysine and serine was observed in the treated *Xcc* cells when compared to control cells.

In *E.coli*, amino acid synthesis pathway is known to be very sensitive to oxidative stress as enzymes involved in amino acid biosynthesis are susceptible to oxidative damage (Mih et al., 2020). It can also represent the intensified use of these amino acids towards mediating a response to TCNP treatment. Amino acids are considered as a sole resource of carbon, nitrogen and protein biosynthesis and essential for bacterial growth and survival (Saldanha et al., 2020; Picchi et al., 2021). Glutamine forms an important source of nitrogen in bacterial cells and is needed for the synthesis of many nitrogen containing compounds, including amino acids (Krysenko and Wohlleben, 2022). Leucine, valine and isoleucine are the three essential branched chain amino acids which are required for the growth of bacteria. Of these, both valine and leucine showed reduction in TCNP treated cells. The observed decrease in the amino acid pool could explain the significant reduction seen in cell growth of TCNP treated *Xcc* cells. Extensive changes in amino acids amount were also observed in N-acetylcysteine treated *Xanthomonas citri* species (Picchi et al., 2021).

Lipids are diverse class of metabolites required for various vital biological functions, especially in the formation of cell membrane. Bacterial membranes are generally composed of lipids mostly phospholipids. During stress, the membrane lipid and fatty acid content in bacteria gets modulated to maintain the membrane fluidity and stability (Cheng et al., 2022). *X. campestris* membranes consist of cardiolipin CL, phosphatidylglycerol PG, phosphatidylethanolamine PE, the methylated PE derivatives monomethyl-PE (MMPE) and phosphatidylcholine (PC). Lysophosphatidylcholine (LPC) serves as a substrate for PC formation in Xanthomonas (Moser et al., 2014). High levels of PC and LPC lipids suggest significant remodelling of membranes following TCNP treatment in *Xcc* cells. Lysophospholipids (LPLs) are known to be generated as metabolic intermediates in bacterial phospholipid turnover or from membrane degradation and gets accumulated in stressed bacteria (Moser et al., 2014; Zheng et al., 2017). A distinct change was also observed in the fatty acid composition of TCNP treated *Xcc* cells. A significant increase in the levels of unsaturated fatty acids, linoleic acid, palmitoleic acid, and oleic acid and saturated fatty acids like stearic acid, palmitic acid was seen in TCNP treated *Xcc* cells. Alteration in the composition of fatty acids, which in turn affects membrane fluidity, is an important adaptive response of bacteria against membrane active compounds (Murínová and Dercová, 2014). Changes in the ratio of long chain to short chain fatty acids has been observed in bacteria under stress conditions to regulate membrane fluidity (Fozo and Quivey Jr., 2003; Chiou et al., 2004). Therefore, changes in the lipids and fatty acid composition in the membrane of TCNP treated *Xcc* cells reflect the stress response from *Xcc* cells to maintain membrane integrity due to the membrane damage upon exposure to TCNPs.

Glutathione (GSH) is an important scavenger of ROS species hence plays a major role in cellular defence response against oxidative stress (Masip et al., 2006). An increase in the levels of glutathione was seen in TCNP treated *Xcc* cells indicating generation of oxidative stress. In H_2_O_2_ treated *Staphylococcus aureus* and *Pseudomonas aeruginosa,* similar accumulation of reduced GSH was observed, which aided in stress resistance (Deng et al., 2014). These observations with TCNP treated *Xcc* cells are also consistent with the studies reported in Liu et al. (2017).Generation of GSH corroborates the ROS generation observed in *Xcc* cells when exposed to TCNPs. GSH is derived from glutamate, cysteine and glycine. A significant decrease in the levels of glutamine was observed in treated *Xcc* cells suggesting its use in glutathione biosynthesis.

Accumulation of purine nucleotides like guanosine monophosphate, inosine monophosphate and pyrimidine nucleoside cytidine in *Xcc* cells exposed to TCNPs suggests altered purine and pyrimidine metabolism. Nucleotide metabolism pathway is crucial for DNA replication, RNA synthesis and for supply of energy needs for cellular proliferation. Alterations in nucleotide pathway indicates change in DNA/RNA turn over and cell growth (Diehl et al., 2022). Changes to nucleotide biosynthesis pathway is observed under different stress conditions in bacteria (Jozefczuk et al., 2010). When confronting stress conditions like oxidative stress, bacterial cells are known to inhibit growth to activate defence-signalling pathways to remove ROS. Build-up of nucleobases and nucleosides are also known to occur in *E. coli* when cells are starving or aging and RNA and DNA demands are low. High concentration of cytidine was observed in aging bacteria and found to impact global transcription as a general stress response (Pan et al., 2021). Therefore, the observed changes in nucleotide metabolism in the TCNP treated *Xcc* cells probably reflects the response to damage to nucleic acid due to ROS or the growth arrest induced by stress.

**Figure 7:**
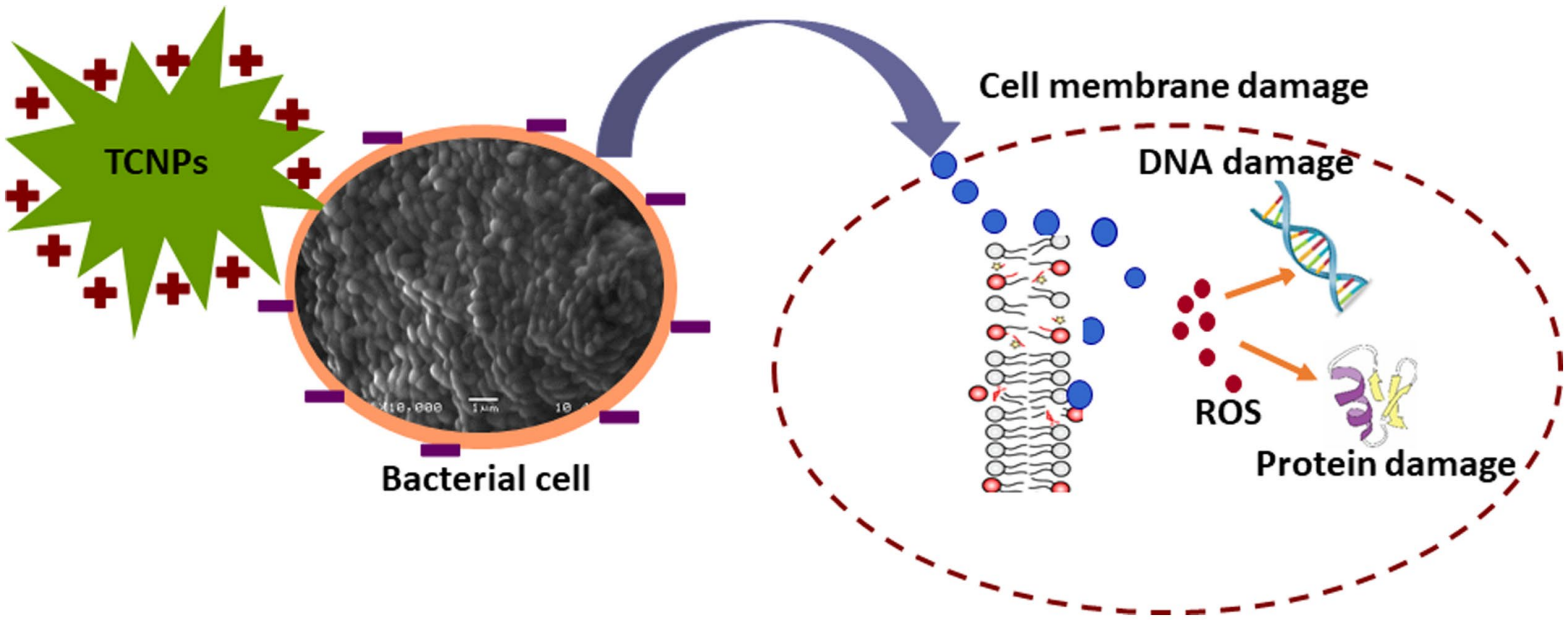
Schematic representation of TCNPs leading membrane disruption and a synergy of action leading to induction of cytotoxicity.

Threonylcarbamoyladenosine (t6A) is a universally conserved nucleoside modification found in transfer RNA (tRNA) (Thiaville et al., 2015). In order to adapt to oxidative stress, bacteria reduce the rate of translation by tRNA degradation (Zhong et al., 2015; Leiva et al., 2020). The accumulation of threonylcarbamoyladenosine in TCNP treated *Xcc* cells probably might be due to oxidative stress-induced global translational regulation of tRNA synthesis. Nicotinamide adenine dinucleotide (NAD ^+^) is a vital metabolite acting as co -factor in several core metabolic redox reactions. NAD^+^ can be synthesized *de novo* from amino acid precursors or from intermediates such as nicotinamide riboside *via* salvage pathway in *E.coli* (Dong et al., 2014). NAD^+^ homeostasis is carefully maintained in cell as disruption of NAD+ synthesis can cause growth arrest and cell death (Wang et al., 2022) Depletion of ribosyl nicotinate suggests alteration of the salvage pathway probably affecting NAD^+^ generation in TCNP treated *Xcc* cells contributing to growth arrest. Carbohydrate metabolism pathway provides carbon and energy to bacteria and alteration to carbohydrate flux is observed in response to oxidative stress (Nikel et al., 2021; Pan et al., 2021). *Xcc* is known to possess fructose metabolism pathway (de Crécy-Lagard et al., 1991). A decrease in endogenous level of fructose in TCNP treated *Xcc* cells indicates differential utilization of sugars and perturbation in carbohydrate metabolism. Overall, the results show that *Xcc* reacts to TCNPs exposure by altering general metabolic pathways mainly amino acids, lipids, nucleotides, and carbohydrate pathways resulting in metabolic reprogramming. These pathways are known to be affected by membrane damage and oxidative stress. These changes agree with ROS generation and lipid peroxidation as observed in TCNP exposed *Xcc* cells. FTIR also revealed changes in similar class of compounds as observed in metabolomic analysis. Therefore, TCNPs exposure resulted in metabolic reprogramming altering amino acids, lipids, proteins and carbohydrates. Taken together TCNPs, distrupts the cell membrane leading to membrane potential alteration and depolarization. This causes penetration of bacterial cell membrane and induction of intracellular antibacterial effects which is explained as shown in the schematic model (Figure 7).

## Conclusion

The present study analysed the metabolic response in *Xcc* cells induced by TCNPs through FTIR and mass spectrometry to elucidate the toxicity mechanism of TCNPs. Physiological assays performed revealed that TCNP’s exposure leads to alteration of membrane potential, ROS generation and lipid peroxidation in *Xcc* cells. FTIR and non targeted metabolomics of *Xcc* cells exposed to sub lethal concentration of TCNPs revealed changes in abundance of metabolites belonging to amino acids, lipids, fatty acids, nucleic acids, carbohydrate and antioxidants. Modification in these classes of metabolites is known to occur as a response to membrane damage and ROS generated oxidative stress. Taken together, the physiological assays and metabolomics studies suggest that TCNPs interacts with membrane, leading to membrane depolarization, ROS generation and changes in membrane permeability. This facilitates the entry of TCNPs into the cells where it can further interact with macromolecules like proteins and nucleic acids possibly generating additional ROS which eventually leads to cell death. This study demonstrates the mechanistic basis of antibacterial activity of TCNPs against important plant pathogen *Xcc* and paves way for the development of eco-friendly crop protection strategies in future.

## Data availability statement

The datasets presented in this article are not readily available because: All relevant data is contained within the article: The original contributions presented in the study are included in the article/Supplementary material, further inquiries and request to access the datasets should be directed to SR, rajanis@tll.org.sg.

## Author contributions

SR and SS conceptualized and designed the study and revised the manuscript and reviewed the final manuscript. SS and NK carried out the experimental work. SS analyzed and interpreted the data and drafted the manuscript. All authors contributed to the article and approved the submitted version.

## Funding

We acknowledge the support from Temasek Life Sciences Laboratory, Singapore.

## Conflict of interest

The authors declare that the research was conducted in the absence of any commercial or financial relationships that could be construed as a potential conflict of interest.

## Publisher’s note

All claims expressed in this article are solely those of the authors and do not necessarily represent those of their affiliated organizations, or those of the publisher, the editors and the reviewers. Any product that may be evaluated in this article, or claim that may be made by its manufacturer, is not guaranteed or endorsed by the publisher.

## Supplementary material

The Supplementary material for this article can be found online at: https://www.frontiersin.org/articles/10.3389/fmicb.2022.1085113/full#supplementary-material
