# Supplementary material for: Biological effects of Thymol loaded chitosan nanoparticles (TCNPs) on bacterial plant pathogen Xanthomonas campestris pv. campestris
Source: Front Microbiol. 2022 Dec 22;13:1085113. doi: 10.3389/fmicb.2022.1085113 (PMC9815552; doi:10.3389/fmicb.2022.1085113)
Supplement: Supplementary file 1 [file Data_Sheet_1.docx]

Supplementary Table S1. Significant metabolites (*****p<0.05) annotated from untargeted metabolomics of TCNPs treated *Xcc*

*
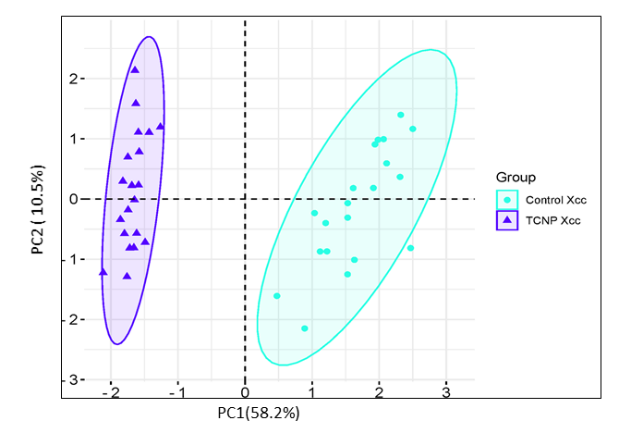
*

Supplementary figure S1: Principal component analysis (PCA) for samples in untargeted metabolomics.
